# Combining of Oncolytic Virotherapy and Other Immunotherapeutic Approaches in Cancer: A Powerful Functionalization Tactic

**DOI:** 10.1002/gch2.202200094

**Published:** 2022-10-20

**Authors:** Hai Zou, Xiao‐Zhou Mou, Biao Zhu

**Affiliations:** ^1^ Department of Critical Care Fudan University Shanghai Cancer Center Shanghai 200032 China; ^2^ Department of Oncology Shanghai Medical College Fudan University Shanghai 200032 China; ^3^ General Surgery Cancer Center Department of Hepatobiliary and Pancreatic Surgery and Minimally Invasive Surgery Zhejiang Provincial People's Hospital (Affiliated People's Hospital of Hangzhou Medical College) Hangzhou 310014 China; ^4^ Key Laboratory of Cancer Molecular Diagnosis and Individualized Therapy of Zhejiang Province Zhejiang Provincial People's Hospital Affiliated People's Hospital of Hangzhou Medical College Hangzhou 310014 China

**Keywords:** cancer, immunotherapy, oncolytic viruses, virotherapy

## Abstract

Oncolytic viruses have found a good place in the treatment of cancer. Administering oncolytic viruses directly or by applying genetic changes can be effective in cancer treatment through the lysis of tumor cells and, in some cases, by inducing immune system responses. Moreover, oncolytic viruses induce antitumor immune responses via releasing tumor antigens in the tumor microenvironment (TME) and affect tumor cell growth and metabolism. Despite the success of virotherapy in cancer therapies, there are several challenges and limitations, such as immunosuppressive TME, lack of effective penetration into tumor tissue, low efficiency in hypoxia, antiviral immune responses, and off‐targeting. Evidence suggests that oncolytic viruses combined with cancer immunotherapy‐based methods such as immune checkpoint inhibitors and adoptive cell therapies can effectively overcome these challenges. This review summarizes the latest data on the use of oncolytic viruses for the treatment of cancer and the challenges of this method. Additionally, the effectiveness of mono, dual, and triple therapies using oncolytic viruses and other anticancer agents has been discussed based on the latest findings.

## Graphical abstract

Combining oncolytic viruses with anti‐checkpoint antibodies such as anti‐PD‐1, anti‐PDL‐1, and anti‐CTLA4 or CAR‐T cells can be effective in cancer therapy in a synergistic fashion. Oncolytic virotherapy induces the expression of PD‐1 and PDL‐1 in the tumor microenvironment components. Virotherapy promotes the infiltration of CD4+ and CD8+ T cells into the tumor tissue.

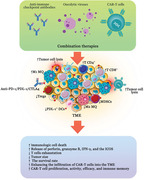

## 1 Introduction

Viruses are intelligent microorganisms that have evolved along with the immune system and have continuously improved their escape mechanisms to the immune system.^[^ ^1^, ^2^, ^3^ ^]^ Some viruses have oncolytic properties that can infect tumor cells and lead to direct lysis of these cells.^[^ ^4^ ^]^ In addition, oncolytic viruses can stimulate immune responses. The most studied oncolytic viruses are Newcastle disease viruses, herpes viruses, coxsackievirus, measles viruses, adenoviruses, polioviruses, poxviruses, and reoviruses.^[^ ^5^, ^6^ ^]^ Therefore, oncolytic viruses can be a moral treatment option in cancer therapy. The unique feature of oncolytic viruses is that they act selectively and remove tumor cells without damaging non‐cancerous cells.^[^ ^7^ ^]^ Initially, oncolytic virotherapy was studied as monotherapy with unsatisfactory therapeutic effectiveness. Several mechanisms, including the immunosuppressive tumor microenvironment (TME), the off‐target sequestration and replication, the existence of neutralizing antibodies, and the balance between viral replication and induced immune response, contribute to oncolytic virotherapy monotherapy resistance.^[^ ^8^ ^]^ Therefore, researchers have used various combination therapies to overcome these barriers using oncolytic viruses and immunotherapy.

Cancer immunotherapy, including methods such as adoptive T cell therapy, immune checkpoint blockade, and monoclonal or bispecific antibodies, has recently succeeded in treating cancer and other immune‐related diseases such as autoimmunity.^[^ ^9^, ^10^, ^11^ ^]^ Nevertheless, it still seems that cancer monotherapy cannot be very effective compared to various combination therapies.^[^ ^12^ ^]^ For instance, a recent study reported that due to the immunosuppressive and immune‐privileged features of glioblastoma, the efficacy of monotherapy with anti‐programmed cell death‐1 (PD‐1)/programmed cell death ligand‐1 (PD‐L1) antibodies was markedly reduced.^[^ ^13^ ^]^ It has been reported that combinations of ipilimumab (an anti‐immune checkpoint antibody) with oncolytic viruses armed with cytokines have promising therapeutic outcomes for unresectable tumors or metastatic.^[^ ^14^, ^15^, ^16^ ^]^ Additionally, experimental studies showed that combining anti HER2/neu chimeric antigen receptor (CAR) T‐cell and oncolytic adenovirus expressing anti‐PD‐L1 and IL‐2 significantly enhanced the survival of animals than CAR‐T cell or oncolytic virus alone.^[^ ^17^ ^]^ Therefore, these types of combination therapies can effectively treat cancer and the need to study and examine the different types of therapeutic regimes using oncolytic viruses and other immunotherapeutic methods is felt to achieve optimal antitumor responses.

This review summarized the most recent data about oncolytic virotherapy in cancer and the challenges with this approach. Moreover, the effectiveness of mono, dual, and triple therapies utilizing oncolytic viruses and other anticancer agents has also been discussed.

## 2 Oncolytic Virus Therapy

Virotherapy has been a treatment option for cancer since the 19th century. However, this therapeutic method has been associated with various limitations, such as tumor cell resistance, off‐target toxicity, immune system responses to the viruses, and the challenges of genetic modification in oncological viruses with genetic engineering.^[^ ^18^ ^]^ In fact, genetic engineering is used to modify the viral genome so that these viruses can efficiently replicate and lysis tumor cells by inducing the expression of specific genes by infected cells, which sometimes are associated with promoting antitumor responses.^[^ ^19^ ^]^ Virotherapy is now considered cancer immunotherapy because it can cause tumor antigen release following cell lysis and elicit immune responses.^[^ ^20^ ^]^ In this context, it has been demonstrated that inserting specific genes in oncolytic viruses such as herpesvirus type 1 (HSV‐1) enhances tumor antigen‐presentation to effector T cells, eliminating tumor cells.^[^ ^21^ ^]^ Talimogene laherperepvec (T‐VEC) is a modified oncolytic HSV‐1 to produce granulocyte‐macrophage colony‐stimulating factor (GM‐CSF) and induce systemic and local antitumor immune responses. T‐VEC was approved by the US food and drug administration (FDA) in 2015 for melanoma treatment.^[^ ^22^ ^]^ It appears that among the wide range of oncolytic viruses studied, the poxviruses family has been used more due to their suitable therapeutic properties. For example, the oncolytic myxoma virus (MYXV), a member of the poxviruses, can infect rabbits but has no pathologic effect on human cells.^[^ ^23^ ^]^ Other oncolytic viruses have been studied in some countries. For instance, oncorine is a modified type 5 human adenovirus (HAdV‐C5) designed for replicating cells with p53 impairment and used to treat head and neck squamous cell carcinoma (HNSCC). In this oncolytic virus, the E1B‐55KD and E3 regions were removed, and this genetic modification has led to enhance the infection of target cells.^[^ ^18^ ^]^

Nevertheless, oncolytic viruses do not always undergo genetic modification. For example, RIGVIR, a strain from the *Picornaviridae* family, has been used to treat melanoma without genetic modification.^[^ ^24^, ^25^ ^]^ As mentioned, the most investigated oncolytic viruses used in preclinical and clinical studies to treat cancer are HSVs, adenoviruses, coxsackieviruses, measles viruses, Newcastle disease viruses, reoviruses, poxviruses, and polioviruses.^[^ ^5^ ^]^ Therefore, oncolytic viruses can be used as intelligent microorganisms to treat cancer. However, various efforts are underway to optimize virotherapy in cancer through genetic modifications and combination therapies to overcome challenges.

## 3 Oncolytic Viruses Mechanisms of Action

Based on available knowledge, oncolytic viruses could exert their antitumor effect via direct tumor cell lysis and stimulation of antitumor immune responses described in this section.

### 3.1 Direct Tumor Cell Lysis

Oncolysis is the term used to describe the process by which oncolytic viruses preferentially infect tumor cells, replicate, and then kill the affected cells via inducing the apoptotic pathways in tumor cells. This process also occurs in adjacent tumor cells and increases the lytic cycle and virus replication.^[^ ^26^, ^27^ ^]^ This lytic cycle usually decreases and eventually ends with the depletion of host target cells and the peak of antiviral immune responses.^[^ ^28^ ^]^ Arming oncolytic viruses refers to inserting genes encoding molecules that amplify oncolysis efficacy. For instance, by arming an oncolytic adenovirus designed to express TNF‐related apoptosis‐inducing ligand (TRAIL), the overexpression of TRAIL increases the oncolytic potency of adenovirus via promoting apoptotic cell death in vitro and in vivo.^[^ ^29^, ^30^ ^]^ TRAIL causes apoptosis primarily in cancer cells through ligation to specific death receptors.^[^ ^31^ ^]^ The unique feature of oncolytic virotherapy is the lysis of tumor cells without damage to normal cells, which can make this method a targeted treatment in cancer therapy.^[^ ^32^ ^]^

Another benefit of oncolytic viruses is their effect on other tumorigenic mechanisms like angiogenesis. The mechanism of the antiangiogenic effects of oncolytic viruses is related to infection of both tumor cells and tumor endothelium, disrupting tumor vasculature.^[^ ^33^ ^]^ However, some studies have proposed the theory that the induction of interferon‐γ (IFN‐γ)‐mediated immune responses can also be effective in inhibiting tumor angiogenesis.^[^ ^34^ ^]^ Following inhibition of angiogenesis, these viruses cause hypoxia, and the tumor cells lack access to oxygen and nutrients, which ultimately reduces tumor growth and progression.^[^ ^35^, ^36^, ^37^ ^]^

### 3.2 Boosting Antitumor Immune Function

The second mechanism, or induction of antitumor responses by oncolytic viruses, usually initiates subsequent tumor cell apoptosis and oncolysis because the immune sensors recognize the danger‐associated molecular patterns (DAMPs) and pathogen‐associated molecular patterns (PAMPs), releasing inflammatory mediators, and enhancing antitumor responses against remained uninfected tumor cells.^[^ ^6^, ^38^ ^]^ As previously mentioned, genetic modifications in oncolytic viruses can have several satisfactory consequences. For example, inserting the genes of cytokines, chemokines, and growth factors into the virus genome can increase the expression of these mediators in the TME to enhance the antitumor immune responses induced by tumor cell lysis. The GM‐CSF is a prime example of this genetic manipulation. The GM‐CSF gene in oncolytic viruses converts them to an immune booster, resulting in overexpression of GM‐CSF by tumor cells. The overproduction of GM‐CSF leads to the maturation and expansion of antigen‐presenting cells (APCs) and the activation of cytotoxic cells.^[^ ^39^ ^]^ Another important genetic modification in oncolytic viruses enhances immune responses by inserting the heat shock protein family A (Hsp70) member 1A (*HSPA1A*) gene.

Further expression of this protein can increase the frequency of NK cells as well as CD4^+^ and CD8^+^ T cells via increasing the delivery of intracellular antigen to the proteasome and the presentation of antigen, improving antitumor responses.^[^ ^40^ ^]^ Interleukin (IL)‐12 is known as an effective anticancer cytokine involved in Th1 differentiation, T‐cell‐mediated tumor cell killing, and tumor angiogenesis inhibition. Since systemic IL‐12 therapy accompanies significant toxicity in humans, employing oncolytic viruses, specially HSVs (OHSV‐IL12), could be a potential therapeutic approach to the local release of IL‐12 in the TME without toxicity.^[^ ^41^ ^]^

Recently, it has been reported that *CCL5*‐armed oncolytic vaccinia viruses induce the expression of CCL5 by infected cells and enhance CCR5‐engineered NK cell infiltration, promoting antitumor responses.^[^ ^42^ ^]^ Adenoviruses are widely used in virotherapy because of their properties. These inherently immunogenic viruses can carry large transgenes, be produced on a large scale, and are well tolerated in the patient's body. TILT‐123 is one of these adenoviruses, which contains the *IL‐2* and tumor necrosis factor‐alpha (*TNF‐α*) genes and induces the expression of these cytokines in the target cells to reinvigorate effector T cells and enhance antitumor response in the TME. Moreover, TILT‐123 is quickly cleared from healthy tissues without damaging vital organs.^[^ ^43^ ^]^ Therefore, genetic modification led to promoting the effectiveness of oncolytic virotherapy.

## 4 Boundaries of Virotherapy

**Figure 1:**
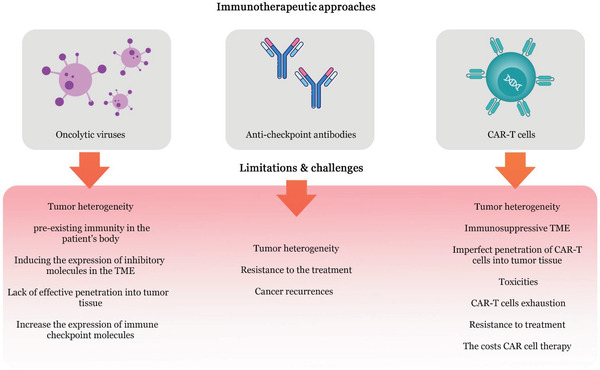
The challenges and limitations of immunotherapeutic approaches. With immunotherapy‐based methods such as virotherapy, immune checkpoint blockade, and CAR‐T cell therapy, cancer monotherapy is usually ineffective because these methods have weaknesses that affect treatment outcomes.

Despite the benefits of virotherapy for cancer treatment, this method is associated with challenges and limitations, briefly discussed in this section (**Figure** **1**).

### 4.1 Ineffective Penetration of Oncolytic Viruses into Tumor Tissue

One of the major problems in virotherapy is the ineffective penetration of these viruses into tumor tissue due to intracellular junctions of epithelial cells and extracellular matrix (ECM) in tumor tissue, leading to treatment resistance.^[^ ^44^, ^45^ ^]^ Studies have shown that following metastasis and the formation of mechanisms such as epithelial to mesenchymal transitions (EMT) and then mesenchymal to epithelial transition (MET), the strength of intercellular junctions increases, and the penetration of oncolytic viruses becomes difficult.^[^ ^46^, ^47^ ^]^ Although in the early stages of infection and before oncolysis, some oncolytic viruses, such as a group of adenoviruses, can partially overcome these epithelial junctions by releasing Pantone‐dodecahedron (Pt‐Dd). However, non‐Pt‐Dd adenoviruses such as HAdV‐C5, more commonly used in virotherapy, are more likely to overproduce fiber protein in the early stages of infection.^[^ ^48^, ^49^ ^]^ To overcome this problem and improve the penetration of oncolytic viruses into tumor tissue, researchers use pretreatment of tumor cells with concomitant administration of hyaluronidase or collagenase with virotherapy, which increases the effectiveness of the treatment.^[^ ^50^ ^]^ Genetic manipulation and integration of the matrix metalloproteinase‐1 (*MMP‐1*) and *MMP‐8* genes into the viral genome can also induce the expression of these proteins and degrade tumor‐associated sulfated glycosaminoglycans, which ultimately improve virus penetration and spread. Correspondingly, apoptosis of tumor cells leads to the formation of channel‐like structures and void spaces in tumor cells, which increases the spread of oncolytic viruses.^[^ ^51^, ^52^ ^]^

### 4.2 Immunosuppressive TME

The infiltration of exhausted cytotoxic T cells and high frequency of regulatory T‐cells (Tregs), myeloid‐derived suppressor cells (MDSC), M2‐macrophages, and tolerogenic DCs induce immunosuppressive milieu through the expression of inhibitory ligands and release of inhibitory mediators such as IL‐10, IL‐27, IL‐35, and tumor growth factor (TGF)‐β.^[^ ^53^ ^]^ This milieu could be a challenge in oncolytic virotherapy. Moreover, hypoxia is a phenomenon that occurs in the TME of solid tumors and leads to stimulation of angiogenesis and dysfunction of antitumor responses.^[^ ^54^ ^]^ The effect of hypoxia on oncolytic viruses is different, and, in some cases, it can reduce the proliferation of oncolytic viruses in tumor cells and disrupt the lytic cycle.^[^ ^55^, ^56^ ^]^ Researchers have designed an oncolytic adenovirus to overcome hypoxia, in which a minimal dual‐specificity promoter that responds to hypoxia and estrogens regulates the expression of the *E1A* gene. This genetic modification improved the replication of the virus in hypoxia.^[^ ^57^ ^]^ Hypoxia‐inducing factor 1 (HIF‐1α) is an essential factor in hypoxia that can enhance angiogenesis and tumor cell metastasis.^[^ ^58^ ^]^ Moreover, it has been reported that HIF‐1α improves HSV‐1 proliferation.^[^ ^59^, ^60^ ^]^ In contrast, HIF‐1α and the expression of its target genes are negatively regulated in hypoxic conditions by the Newcastle disease virus.^[^ ^61^ ^]^ Tumor resistance to oncolytic viruses is another problem with virotherapy. It has been demonstrated that sodium orthovanadate (vanadate) through the epidermal growth factor receptor (EGFR) pathway can overcome tumor resistance to these viruses and improve the antitumor effects of RNA‐based oncolytic viruses in a synergistic fashion.^[^ ^62^ ^]^

### 4.3 Immune System and Antiviral Responses

The immune system and its components are another challenges in virotherapy because the immune system is designed to clear pathogen microorganisms. Pre‐existing neutralizing antibodies, effector immune cells, and increased IFNs following a viral infection or immunization can decrease virotherapy effectiveness. To address this problem, masking oncolytic viruses with polymers or other materials and using cellular carriers as delivery vehicles can increase their half‐life in a protective manner.^[^ ^63^, ^64^ ^]^ Furthermore, it has been revealed that using valproic acid, a histone deacetylase (HDAC) inhibitor, can figure out epigenetic modifications to inhibit the expression of IFNs.^[^ ^65^, ^66^ ^]^ However, despite improving the proliferation of oncolytic viruses, valproic acid inhibits the migration and infiltration of NK cells and M1 macrophages into the TME and prevents tumor cell apoptosis.^[^ ^67^ ^]^ Another antiviral mechanism of the immune system is the production of RNase L, which targets and destroys the virus's RNA. The use of inhibitors of this enzyme, such as sunitinib, can partially compensate for this problem.^[^ ^68^, ^69^ ^]^ In general, suppressing the immune system and its components is a relatively decent solution to protect against the destruction of oncolytic viruses, which eventually increases the effectiveness of treatment. In this context, cytokine therapy (tumor growth factor‐β [TGF‐β]), inhibition of angiogenic factors as well as cyclophosphamide as an immunosuppressant can be beneficial.^[^ ^70^, ^71^, ^72^, ^73^ ^]^ However, it should be noted that suppressing the immune system also affects its antitumor responses or tumor progression, which needs further studies.

Based on previous lessons from therapeutic strategies in cancer, mutations in tumor cells and tumor heterogenicity are other hindrances in oncolytic virotherapy.^[^ ^74^ ^]^

### 4.4 Diagnosis, Monitoring, and Treatment Challenges

Following treatment with oncolytic viruses, it has been revealed that these viruses have little tropism in tumor cells or insufficient delivery of viruses to the tumor site, reducing the effectiveness of treatment in clinical settings.^[^ ^75^, ^76^ ^]^ So far, various strategies have been proposed to solve this problem, such as modifications in the surface of viruses by adding bispecific adapters or tripeptide motifs and using single‐chain variable fragment (scFv) to improve the binding of the viruses to the tumor cell.^[^ ^39^, ^77^, ^78^, ^79^, ^80^, ^81^, ^82^, ^83^, ^84^, ^85^, ^86^, ^87^ ^]^ For instance, in tumor models, it has been demonstrated that insertion of a tripeptide Arg‐Gly‐Asp (RGD) motif into the HI loop of the adenovirus fiber knob domain dramatically improves infection efficiency and lethal effect by inhibiting autophagy and promoting apoptosis.^[^ ^77^ ^]^ Here is a brief explanation to better understand these molecules’ functions. An adapter is a bispecific binding molecule consisting of two modules, one of which binds to the virus’ coat and the other attaches to a specific cell‐surface receptor on the target cell. Adapters are easily scalable for clinical trials.^[^ ^88^ ^]^ Moreover, an scFv is a fusion protein of the variable sections of the heavy and light chains of immunoglobulins, joined by a short linker peptide of ten to twenty‐five amino acids.^[^ ^89^ ^]^ In addition, some serotypes of oncolytic viruses that can bind to the surface components of tumor cells, such as sialic acid, can prevent oncolytic viruses from being “off‐target.”^[^ ^90^ ^]^

On the other hand, the lack of appreciated biomarkers to approve the response of patients with cancer to virotherapy is another challenge of this therapeutic approach. However, some biomarkers, such as human inhibitory receptors Ig‐like transcript 2 (ILT2) and high mobility group box‐1 (HMGB‐1), have been suggested as treatment monitoring biomarkers.^[^ ^91^, ^92^ ^]^

Moreover, studies have established the viability of regulating oncolytic virus tropism by inserting synthetic target sequences for differently produced microRNAs (miRNAs) into the virus genome.^[^ ^93^, ^94^ ^]^ By selecting miRNA species whose expression is strongly upregulated in healthy tissues but downregulated in tumor cells, viral replication and toxicity can be significantly reduced in off‐target tissues. This post‐entry (de‐)targeting technique is, in principle, adaptable to any virus, regardless of the nature of its nucleic acid or virus structure. Furthermore, the small miRNA target sites (miRTS) permit perfect viral replication kinetics and application in viruses with tightly restricted genome sizes.^[^ ^95^ ^]^ It has demonstrated that combining target sites for distinct miRNAs inserted into a single viral genome protects several sensitive tissues, which is vital for systemic virotherapy methods.^[^ ^96^ ^]^

## 5 Cancer Immunotherapy

Many advances in cancer immunotherapy have been made in recent years due to the apparent interaction between the immune system and tumors. However, these methods have not yet been able to significantly increase survival rates in some solid tumors, especially in advanced stages. Several factors can negatively affect cancer immunotherapy, including immunosuppressive TME, hypoxia, high acidity, lack of effective penetration into tumor tissue, and other unknown factors.^[^ ^97^ ^]^ The most important methods of immunotherapy include immune checkpoint blockade, adoptive cell therapy, and monoclonal antibodies or their derivatives, each of which is briefly discussed in this section (Figure 1).

### 5.1 Immune Checkpoint Blockade

Immune checkpoints are a normal part of the immune system, involved in homeostasis and preventing dysregulated and destructive immune responses to protect normal cells and tissues. Immune checkpoint proteins such as PD‐L1 on tumor cells and PD‐1 on T cells are involved in immunosuppressive mechanisms. Following the ligation of these ligands and receptors, the “off” signal is transmitted to effector T cells, inducing the contraction of the immune responses.^[^ ^98^ ^]^ Tumors usually seize this opportunity by expressing these inhibitory molecules, hindering antitumor responses and facilitating the growth and development of tumor cells.^[^ ^99^ ^]^ Therefore, researchers have developed drugs that can bind to these inhibitory molecules and suppress the “off” signal transmission to T cells, killing tumor cells.^[^ ^100^ ^]^ The US food and drug administration (FDA) approved using immune checkpoint inhibitors to treat various human malignancies with promising clinical outcomes.^[^ ^101^ ^]^ In 2011, the FDA approved ipilimumab (anti‐cytotoxic T‐lymphocyte associated protein 4 [CTLA4]) for the treatment of metastatic melanoma, after which other checkpoint inhibitors targeting the PD‐1/PD‐L1 axis have been used to treat several tumors and approved by the FDA.^[^ ^102^ ^]^ Although patients treated with immune checkpoint inhibitors usually respond well at the beginning of treatment, they become resistant after a while and experience recurrences.^[^ ^103^ ^]^ For this reason, combination therapy is commonly used to administer immune checkpoint inhibitors and other anticancer agents simultaneously. A phase 1 dose‐escalation trial (NCT05222932) has been designed to evaluate the safety of a combination of the TILT‐123 (oncolytic adenovirus) with an anti‐immune checkpoint antibody (avelumab, anti‐PD‐L1) in patients with advanced solid tumors refractory to or progressing after anti‐PD‐L1. Although this study is in the stage of recruiting patients and its outcomes have not yet been determined.^[^ ^104^ ^]^

Evidence has demonstrated that oncolytic virotherapy administered in the neoadjuvant setting can promote treatment outcomes. Additionally, combining “armed” recombinant viruses such as IL‐12‐armed HSV as neoadjuvant oncolytic virotherapy with immune checkpoint inhibitors could improve the therapeutic viability of neoadjuvant therapy and surgical outcomes. However, numerous questions remain mysterious due to the relatively new interest in engaging oncolytic viruses in neoadjuvant therapy.^[^ ^105^ ^]^

### 5.2 Adoptive T Cell Therapy

Adoptive T cell therapy is a sort of immunotherapy in which T cells are administered to a patient to aid the body in fighting diseases such as cancer.^[^ ^106^ ^]^ T cells are often isolated from the patient's blood or tumor tissue, proliferated in vitro, and then returned to the patient to assist the immune system in fighting cancer. Sometimes, T cells are modified in the laboratory to enhance their ability to target and destroy cancer cells. CAR‐T cell treatment and tumor‐infiltrating lymphocyte (TIL) therapy are examples of adoptive cell therapy.^[^ ^107^ ^]^ T‐cell donor‐based adoptive cell therapy is being investigated to treat some cancers and infections.

Classically, a tumor biopsy is obtained from the patient, undertreatment IL‐2 tumor fragments are grown in vitro, autologous TILs are isolated and expanded, and large quantities of these effectors lymphocytes are re‐infused into the patient.^[^ ^108^ ^]^ In innovative methods, immune cells such as NK and T cells are isolated from cancer patients. After selecting a tumor‐specific antigen, using genetic engineering methods, a CAR consisting of an extracellular domain containing scFv, transmembrane, and intracellular domain containing costimulatory molecule is assembled on the cells. After proliferation and expansion, these powerful cells are re‐infused into the patient's body to identify tumor cells expressing the target antigen.^[^ ^109^ ^]^ These cells’ advantage is that they depend on the major histocompatibility complexes (MHCs) and can detect and respond to tumor cell antigens without antigen presentation by APCs.^[^ ^110^ ^]^ Although this treatment has been very successful in blood malignancies, in solid cancers, CAR cell therapy still faces failures because of the inefficient penetration of these cells into tumor tissue, various toxicities, CAR‐T cells exhaustion, resistance to treatment, as well as the costs of CAR cell therapy.^[^ ^111^, ^112^ ^]^ CAR‐T cells use antibody fragments (scFv) that bind to specific surface proteins on tumor cells. Another outcome of genetic manipulation on T cells is T cell receptor (TCR) T cell therapy. TCR‐T cells can recognize intracellular tumor antigens presented by APCs and are restricted to MHC molecules.^[^ ^113^ ^]^ Therefore TCR‐T cells can recognize a broader range of tumor antigens than CAR‐T cells. Nevertheless, TCR‐T cell therapy is also limited due to its dependence on MHC molecules and antigen presentation by APCs.^[^ ^114^ ^]^

### 5.3 Monoclonal Antibodies and Antibody Derivatives

Using monoclonal antibodies (mAbs) against tumor‐specific antigens is another effective method of immunotherapy. These antibodies are sometimes administered directly and sometimes conjugated with a cytotoxic agent and can deliver the drug to the tumor site effectively and purposefully. On the other hand, the ability to make extensive modifications in the structure of antibodies has made these “magic bullets” a potential option for immunotherapy and other therapeutic approaches.^[^ ^115^ ^]^ As mentioned, using specific anti‐immune checkpoints and engineered antibodies to produce scFvs and CAR cells has been beneficial in treating cancer. Bispecific antibodies also are produced by engineering approaches. These antibodies have a dual affinity for two specific antigens in the TME. An example of bispecific antibodies is catumaxomab, which can simultaneously activate innate immune cells via the fragment crystallizable region (Fc region) and bind to CD3 in the TCR complex and epithelial cell adhesion molecule (EpCAM) is a tumor antigen, inducing a robust antitumor immune response.^[^ ^116^ ^]^

Moreover, bispecific T cell engager (BiTE) molecules are a promising tactic to promote T cell immunity^[^ ^100^ ^]^ directly. It has been demonstrated that BiTEs can target several antigens, such as CD3 and either CD19, EpCAM, or epidermal growth factor receptor (EGFR). In these molecules using genetic engineering, tumor antigen‐targeted scFvs and anti‐CD3 scFvs are connected by a linker. Following the formation of a bridge between the tumor cell and the T cell, which BiTE performs, the T cell is activated and releases cytokines, eventually leading to the killing of the tumor cell. The advantage of BiTEs is that low doses of these bispecific antibodies can stimulate antitumor responses and overcome mutations of signaling pathways responsible for resistance to the treatment.^[^ ^117^ ^]^

Despite the relatively satisfactory outcomes of immunotherapy in cancer treatment, it has been found that monotherapy cannot be effective due to the limitations of each of the mentioned immunotherapy methods, and combination therapies can significantly improve the effectiveness of treatment.

## 6 Combining Oncolytic Viral Therapy with Chemotherapy and Immunotherapy

**Figure 2:**
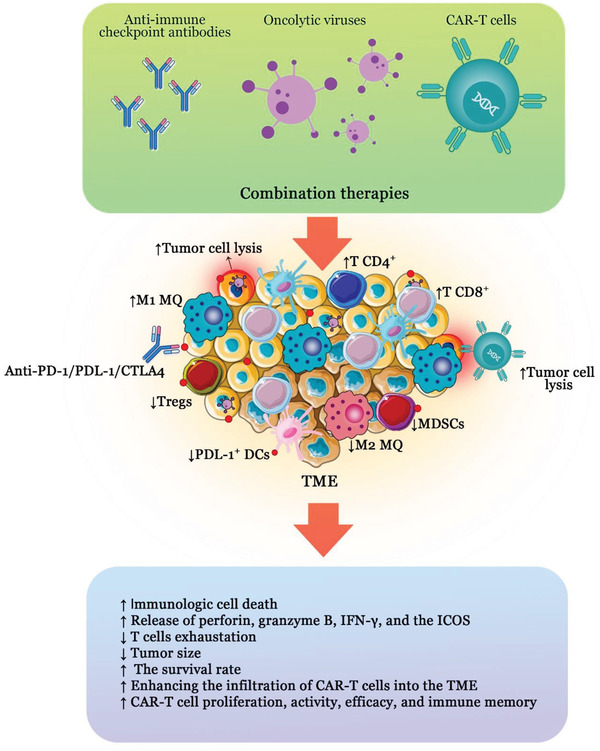
Combination therapy outcomes in cancer. Combining oncolytic viruses with anti‐checkpoint antibodies such as anti‐PD‐1, anti‐PDL‐1, and anti‐CTLA4 or CAR‐T cells can be effective in cancer therapy in a synergistic fashion. Oncolytic virotherapy induces the expression of PD‐1 and PDL‐1 in the TME components. On the other hand, virotherapy promotes the infiltration of CD4+ and CD8+ T cells into the tumor tissue. Therefore, combining anti‐checkpoint antibodies with virotherapy increase the effectiveness of treatment by induction of antitumor responses and reduction of immunosuppressive cell infiltration. Additionally, oncolytic viruses can help CAR‐T cell therapy by enhancing the locomotion and recruitment of CAR‐T cells into the TME as well as promoting proliferation and activation of these engineered T cells.

Cancer monotherapy using oncolytic viruses has low efficacy due to tumor heterogeneity, pre‐existing immunity in the patient's body, expression of inhibitory molecules in the TME, and lack of effective penetration into tumor tissue. Therefore, to increase the effectiveness of treatment and create a synergistic state, researchers use combination therapies with oncolytic viruses and other antitumor agents such as chemotherapy and immunotherapy^[^ ^118^, ^119^ ^]^ (**Figure** **2**). This section discusses the results of the latest studies on combining oncolytic virotherapy with other chemo‐immunotherapeutic regimens for treating various types of human malignancies (**Table** **1**).

**Table 1:** Combination therapies using oncolytic viruses and other immunotherapeutic approaches

Oncolytic virus	Intervention	Type of study	The outcome of combination therapy	Ref.
HSV‐1716	Anti‐PD‐1	Murine rhabdomyosarcoma model	Increasing CD4^+^ and CD8^+^ T cell‐mediated antitumor responses but not Tregs	[141]
VVWR/TK‐RR‐/FCU1	Immune checkpoint blockers or oxaliplatin	MCA205 fibrosarcoma cells/Mouse model	Inducing abscopal effects on distant untreated tumor cells with defects in type I IFN signaling Inducing immunologic cell death	[142]
Vaccinia virus	Anti‐PD‐L1	Ovarian and colon cancer models	Increasing the infiltration of CD4+ and CD8+ T cells Promoting the release of perforin, granzyme B, IFN‐γ, and the ICOS Reducing the frequency of PD‐1^+^CD8^+^ exhausted T cells and virus‐induced PD‐L1^+^ immunosuppressive cells such as Tregs, TAMs, MDSCs, and DCs	[143]
Human reovirus	Anti‐PD‐1	Glioma model	Improve antitumor responses	[127]
VSV‐mIFNβ‐NIS	Anti‐PD‐L1	Murine AML cell line C1498 (ATCC TIB‐49) AML mouse model	Improving antitumor responses and animal survival compared with monotherapy with anti‐PD‐L1 or VSV‐mIFNβ‐NIS Reducing tumor burden in various organs of the studied mice Increasing the frequency of effector CD4^+^ and CD8^+^ TILs in the TME	[146]
T‐VEC	Anti‐PD‐1	Patients with stage IIIC to stage IVM1b melanoma	Increasing the number of CD8^+^ T cells, as well as virus‐induced PD‐L1 expression Suppressing the expression of PD‐L1 Improving immunotherapy's effectiveness through reprogramming the TME	[16, 147]
HSV‐aPD‐1	Anti‐PD‐1	MC38 and B16–F10 models	Promoting T‐cell infiltration and antitumor immune responses more than unloaded viruses or anti‐PD‐1 monotherapies Upregulating antigen presentation and IFN pathway‐related genes Downregulating the expression of cell adhesion and angiogenesis‐associated genes	[149]
VT1093M	VT1092M (Express mouse anti‐PD‐1 antibody)	Murine CT26 colon adenocarcinoma model	Inhibiting primary tumor growth Preventing contralateral untreated tumor growth Generating a vaccine‐like immune response, activate tumor‐specific T cells Prolonging the OS rate of the studied mice	[150]
VACV; JX‐594	Anti‐PD‐1	EMT6, B16‐F10, LLC, MB49, MC38, CT26, and 4T1 cancer cell lines Animal models	Increasing T cell factor‐1(TCF‐1)^+^ stem‐like T Enhancing therapeutic effectiveness. Inducing durable antitumor immunity	[151]
ΔTK‐Armed‐VACV	Expressing anti‐PD‐1 and anti‐4‐1BB antibody genes	A549 or 4T1 tumor‐bearing mouse model	Induced tumor‐specific CD8^+^ T cells Releasing of IFN‐γ Inhibiting tumor growth	[153]
AdV‐D24‐ICOSL‐CD40L	Anti‐PD‐1	MUG, Mel‐1, and MUG Mel‐2 Immunocompetent C57BL/6 melanoma B16V mouse model	Reducing tumor size Increasing the survival rate	[154]
V937	Anti‐CTLA4	Open‐label, phase II study Unresectable stage IIIC or IV melanoma	Inducing systemic immune activation Durable responses (>26 weeks)	[159]
G47Δ	Anti‐CTLA4	Murine esophageal SCC cell lines, AKR and HNM007, SCCVII, Hepa1‐6, murine melanoma cell line B16‐F10, and Vero Animal models	Improving antitumor responses Recruiting of effector T cells into the TME and decreasing the frequency of Tregs at the tumor site Upregulating the expression of various genes involved in inflammatory responses and T cell activation	[160]
RFlu‐CTLA4 rFlu‐huCTLA4	Expressing anti‐CTLA4	H22 mouse model (HCC)	Inhibiting tumor cell growth Prolonging the OS of treated mice Increasing the number of CD4^+^ and CD8^+^ T cells	[161, 162]
M1	Anti‐CTLA4	Melanoma and prostatic tumor models	Inducing tumor suppression and lymphocytotoxicity Decreasing Tregs and increasing the infiltration of CD45^+^ immune cells and CD4^+^ or CD8^+^ T cells Inducing the release of IFN‐γ, perforin, and granzyme Extending the OS rate compared with monotherapy with M1	[163]
G47‐mIL‐12	Anti‐PD‐L1 and anti‐CTLA4 (Triple therapy)	GBM model	Triple therapy was more effective than dual therapy using anti‐CTLA4 and anti‐PD‐1 antibodies to improve the survival of animals	[165]
Adenovirus	Anti‐PD‐L1 and anti‐CTLA4 (Triple therapy)	TNBC model	Inhibiting tumor growth and prolonged survival Recruiting CD8+ T cells, inducing memory T cells Decreasing the number of Tregs and M2 macrophages in the TME	[166]
Ad5D24/CCL5/IL‐15	Anti‐GD2 CAR‐T cell	Neuroblastoma tumor model	Enhancing the infiltration of anti‐GD2 CAR‐T cells into the TME Eliminating tumor cells	[169]
OAd/IL‐2/TNF‐α	meso‐CAR T‐cells	BxPC‐3, Capan‐2, AsPC‐1 KrasLSL.G12D/+p53R172H/+ (KPC) mouse pancreatic tumor model	Expressing IL‐2 and TNF‐α Boosting the functions of mesothelin‐redirected CAR‐T cells	[171]
Adenovirus expressing BiTE targeting EGFR	Anti‐FR‐α CAR‐T cells	SKOV3, HCT116, Panc‐1, NCI‐H226, C30 Animal models	Promoting CAR‐T cells proliferation, activation, and tumor cell killing ability Prolonging the survival of studied mice	[175]
CAdVECPDL1	Anti‐Her2 CAR‐T cells	Human prostate cancer cell line PC‐3, human non‐small cell lung carcinoma cell line A549, human HCC cell line HepG2, and human squamous cell carcinoma line SiHa Animal models	Expressing anti‐PD‐L1 mini‐bodies Enhancing antitumor effect of CAR‐T cells Prolonging the survival rate of the treated animals with CAdVECPDL1 and anti‐Her2 CAR‐T cells compared with monotherapy by CAdVECPDL1 or anti‐Her2 CAR‐T cells	[180]
CAd12/PDL1	CAR‐T cells	HNSCC xenograft model	Expressing anti‐PD‐L1 mini‐bodies and IL‐12p70 Inducing CAR expression and killing of tumor cells by CAR‐T cells	[17]
Oncolytic adenovirus (CAd) expressing IL‐12p70	HER2‐specific CAR‐T cells	The human HNSCC line FaDu and human TNBC cancer cell line MDA‐MB468 Animal model	Encoding epitopes to stimulate naïve TCRs Enhancing CAR‐T cell proliferation, activity, efficacy, and immune memory	[182]

### 6.1 Combinations of Oncolytic Viruses and Chemotherapy Drugs

Preclinical and clinical studies have shown that combining oncolytic viruses with chemotherapy drugs can lead to more effective treatment than using these therapeutic approaches alone.^[^ ^120^, ^121^ ^]^ In this regard, numerous compounds have been examined, the most important of which are cyclophosphamide,^[^ ^73^ ^]^ cisplatin,^[^ ^122^ ^]^ gemcitabine,^[^ ^123^ ^]^ 5‐fluorouracil (5‐FU),^[^ ^124^ ^]^ doxorubicin,^[^ ^125^ ^]^ taxanes,^[^ ^126^ ^]^ histone deacetylase inhibitors,^[^ ^127^ ^]^ rapamycin,^[^ ^128^ ^]^ cyclooxygenase 2 inhibitors,^[^ ^129^ ^]^ EGFR inhibitors,^[^ ^130^ ^]^ and other agents.

Recent studies have shown that combination therapy with oncolytic viruses and chemotherapy can increase the effectiveness of treatment and prevent drug resistance.^[^ ^131^ ^]^ Multidrug‐resistant 1 (MDR1) is involved in chemoresistance and is highly expressed by tumor cells. An investigation designed OBP‐702, a tumor suppressor *p53*‐expressing oncolytic adenovirus, with the capability of inducing antitumor responses against human osteosarcoma cells. This study revealed that the expression of MDR1 was significantly upregulated in doxorubicin‐resistant osteosarcoma cells and suppressed by OBP‐702, inducing doxorubicin‐ apoptosis. Moreover, combining OBP‐702 and DOX could remarkably suppress tumor growth in the doxorubicin‐resistant MNNG/HOS xenograft tumor model than monotherapy.^[^ ^131^ ^]^ Therefore, tumor‐specific virotherapy might be a potential strategy for inhibiting chemoresistance in tumor cells by suppressing the expression of MDR1.

Unlike chemotherapy, immunotherapy treats patients by acting on their immune systems, which directly affects cancerous and normal cells. Immunotherapy can induce the body's immunological response and boost the immune system to recognize and eliminate cancer cells. Although each therapeutic approach has its advantages and disadvantages, both can create a synergistic effect with oncolytic viruses and be effective in cancer therapy.

### 6.2 Combinations of Oncolytic Viruses and Immune Checkpoint Blockers

#### 6.2.1 Anti PD‐1 and Anti PD‐L1 Antibodies

As previously discussed, immune checkpoint blockade is one of the most effective methods of cancer immunotherapy that can enhance antitumor responses by inhibiting inhibitory molecules such as PD‐1, PD‐L1, and CTLA4. The US FDA‐approved antibodies blocking the PD‐L1/PD‐1 axis are cemiplimab, nivolumab, pembrolizumab, atezolizumab, avelumab, and durvalumab.^[^ ^132^, ^133^, ^134^ ^]^ However, there are limitations to using this treatment, and the tumor can become resistant to anti‐inhibitory molecules. The low frequency of CD8^+^ T cells in the TME and tumor escape by the mechanism of downregulating PD‐L1 expression reduces the effectiveness of cancer treatment using immune checkpoint blockers.^[^ ^135^, ^136^, ^137^ ^]^

Therefore, combining the immune checkpoint blockers with other methods, such as virotherapy, can increase the effectiveness of treatment in a synergistic state.^[^ ^138^ ^]^ The combination of virotherapy and immune checkpoint blockers is a win‐win result because immune checkpoint blockers inhibit PD‐L1 induced by oncolytic viruses, and oncolytic viruses cause more infiltration of CD4^+^ and CD8^+^ T cell into the TME and IFN‐γ secretion. For instance, canerpaturev(C‐REV), an attenuated strain of the HSV‐1, exhibits antitumor properties. However, C‐REV can upregulate the expression of PD‐L1 on tumor cells, macrophages, and DCs. It has been reported that the frequency of activated CD8^+^PD‐1^−^ TILs increased in the TME following virotherapy with C‐REV.^[^ ^139^ ^]^ Therefore, combination therapy using oncolytic viruses and immune checkpoint inhibitors dramatically enhances antitumor responses, and the weaknesses of each method alone are covered by the other.^[^ ^118^, ^140^ ^]^ In murine rhabdomyosarcoma models, anti‐PD‐1 and HSV‐1716 were used, and findings demonstrated that this combination could be effective in cancer treatment through increasing CD4^+^ and CD8^+^ T cell‐mediated antitumor responses but not Tregs. Moreover, combining anti‐PD‐1 and HSV‐1716 synergistically induces antitumor responses than HSV‐1716 or anti‐PD‐1 alone.^[^ ^141^ ^]^

Western Reserve strain, an engineered vaccinia virus (VVWR/TK^−^RR^−^/FCU1), can promote immunologic‐mediated tumor cell death and reprogram the components of TME. Moreover, a combination of VVWR/TK^−^RR^−^/FCU1with immune checkpoint blockers or oxaliplatin significantly makes abscopal effects on distant untreated cancer cells. These therapeutic effects are especially evident when tumor cells have defects in type I IFN signaling.^[^ ^142^ ^]^

A study on ovarian and colon cancer models revealed that an oncolytic vaccinia virus could recruit effector T cells into the TME and induce PD‐L1 expression on both immune and tumor cells at the tumor site. Combining oncolytic vaccinia virus and anti‐PD‐L1 increases the infiltration of CD4^+^ and CD8^+^ T cells, promoting the release of perforin, granzyme B, IFN‐γ, and the inducible costimulator (ICOS, CD278). Furthermore, the frequency of PD‐1^+^CD8^+^ exhausted T cells and virus‐induced PD‐L1^+^ immunosuppressive cells such as Tregs, TAMs, MDSCs, and DCs were reduced in the TME. This transformation in the balance of immune responses to antitumor responses can eliminate tumor cells by the immune system components.^[^ ^143^ ^]^ It has been reported that intravenous infusion of oncolytic human reovirus can infect tumor cells and increase the frequency of cytotoxic T cell tumor infiltration in patients with glioma. In this study, the effect of oncolytic viruses on increasing the expression of inhibitory molecules and IFN‐associated genes was observed again. It was shown that combination therapy with reovirus and anti‐PD‐1 could improve antitumor responses.^[^ ^144^ ^]^ Triple‐negative breast cancer (TNBC) is an aggressive human malignancy for which available therapeutic approaches are imperfect and accompany severe toxicities. An animal model study showed that treatment by oncolytic viruses and immune checkpoint blockers could prevent relapse in the majority of the studied animals.^[^ ^145^ ^]^ These data demonstrated that oncolytic viruses could use as an immune checkpoint blocker‐sensitizing approach and neoadjuvant treatment option in cancer.

A study on murine acute myeloid leukemia (AML) models demonstrated that employing oncolytic vesicular stomatitis virus (VSV), which encodes IFNβ and the sodium iodide symporter (NIS) reporter, showed that viral infection could not increase the expression of PD‐L1 on the surface of tumor cells. However, combination therapy by VSV‐mIFNβ‐NIS and anti‐PD‐L1 improved antitumor responses and animal survival compared with monotherapy with anti‐PD‐L1 or VSV‐mIFNβ‐NIS. This combination therapy also reduced tumor burden in various organs of the studied mice and increased the frequency of effector CD4^+^ and CD8^+^ TILs in the TME. Interestingly, this synergistic therapeutic effect has been reported without adverse effects.^[^ ^146^ ^]^ Some clinical trials evaluated the impact of combination therapy with T‐VEC and anti‐PD‐1(pembrolizumab), and the findings showed that in responder patients, the number of CD8^+^ T cells, as well as virus‐induced PD‐L1 expression, were increased. Therefore, the expression of PD‐L1 is suppressed by pembrolizumab. These results propose that combination therapy with oncolytic viruses and immune checkpoint inhibitors may improve immunotherapy's effectiveness through reprogramming the TME.^[^ ^16^, ^147^ ^]^

Some preclinical studies believe that in combination therapies using oncolytic viruses and anti‐PD‐1, the timing of anti‐PD‐1 administration is critical and can influence therapeutic achievement. A study demonstrated that after initial virotherapy, the simultaneous use of anti‐PD‐1 and oncolytic viruses is essential because oncolytic viruses preserve the priming of effector T cells while blocking PD‐1 supports to overcome T cell exhaustion. However, a wide range of confounding factors such as type of tumor, type of oncolytic virus, absence or presence of cytokine transgenes carried by oncolytic viruses, initiation time of treatment and frequencies, dosages, and duration of oncolytic virotherapy and anti‐PD‐1, may influence the validity of findings.^[^ ^148^ ^]^

A recombinant HSV‐1 (HSV‐aPD‐1) capable of carrying and expressing anti‐PD‐1 mAbs in tumor cells was evaluated in combination with anti‐PD‐1 mAbs in cancer treatment. This study showed that HSV‐aPD‐1 promoted T‐cell infiltration and antitumor immune responses more than unloaded viruses or anti‐PD‐1 monotherapies. Additionally, tumor RNAseq analysis revealed that antigen presentation and IFN pathway‐related genes upregulated, whereas the expression of cell adhesion and angiogenesis‐associated genes downregulated following combination therapy.^[^ ^149^ ^]^ VT1093M and VT1092M are two engineered oncolytic viruses that express anti‐PD‐1antibody and IL‐12, respectively. A study on CT26 colon adenocarcinoma murine models showed that both VT1093M and VT1092M could induce antitumor responses. Combining and intratumoral administration of these engineered oncolytic viruses inhibit primary tumor growth, prevent contralateral untreated tumor growth, generate a vaccine‐like immune response, activate tumor‐specific T cells, and extend the overall survival (OS) rate of the studied mice.^[^ ^150^ ^]^ Intratumorally administration of oncolytic vaccinia virus (VACV; JX‐594) effectively amplified the frequency of tumor‐specific CD8^+^ T cells and reduced the infiltration and number of immunosuppressive cells in the TME. It has been reported that the majority of tumor‐specific CD8^+^ T cells are T cell factor‐1(TCF‐1)^+^ stem‐like T cells, and combining JX‐594 with anti‐PD‐1 improved the proliferation of these T cells and enhanced therapeutic effectiveness. Therefore, ready‐to proliferation tumor‐specific CD8^+^ T cells in the bone marrow (BM) and spleen for a prolonged period lead to durable antitumor immunity.^[^ ^151^ ^]^

Recently, an interesting study revealed that combination therapy using oncolytic measles vaccine (MV) vectors encoding anti‐PD‐1 or anti‐PD‐L1 genes and PD‐1/PD‐L1 blockers leads to a slight increase in survival and intratumoral IFN‐γ compared with monotherapy by control MV or anti‐PD‐1/PD‐L1 antibodies.^[^ ^152^ ^]^ Therefore, combining oncolytic viruses encoding cytokine genes (TNF‐α, IL‐2, IL‐12, IFN‐γ) with anti‐PD‐1/PD‐L1 antibodies can be more effective in the treatment than anti‐PD‐1 or anti‐PD‐L1 encoding MVs and anti‐PD‐1/PD‐L1 antibodies. A thymidine kinase (TK) gene deleted oncolytic vaccinia virus (ΔTK‐Armed‐VACV) encoding anti‐PD‐1, and anti‐4‐1BB (CD137) antibody genes were designed to treat cancer. Findings showed that the ΔTK‐Armed‐VACV induced tumor‐specific CD8^+^ T cells, the release of IFN‐γ, and repressed tumor growth in A549 or 4T1 tumor‐bearing mice models.^[^ ^153^ ^]^ Oncolytic adenovirus AdV‐D24‐ICOSL‐CD40L expressing potent costimulatory molecules could be effective in the clinic via inducing antitumor immune responses in melanoma therapy. It has been reported that combining the AdV‐D24‐ICOSL‐CD40L with anti‐PD‐1antibody reduced tumor size and increased survival.^[^ ^154^ ^]^

Taken together, these findings indicated that the combination of oncolytic virotherapy and immune checkpoint blockers such as anti‐PD‐1 or anti‐PD‐L1 could be effective in cancer treatment via suppressing virus‐induced inhibitory molecules, the release of tumor‐specific antigens, and the infiltration of CD4^+^ and CD8^+^ T cells at the tumor site. In this regard, genetic manipulation of oncolytic viruses to express anti‐PD‐1/PD‐L1 antibody genes can also be attractive, although some studies have shown that this method is not more effective than combining oncolytic viruses with anti‐PD‐1 or anti‐PD‐L1 antibodies.

#### 6.2.2 Anti‐CTLA4 Antibodies

As a CTLA4 blocking antibody, Ipilimumab has been approved by the US FDA (March 25, 2011) to treat melanoma.^[^ ^155^ ^]^ However, monotherapy with ipilimumab could lead to immune‐related adverse events (irAEs); therefore, combining oncolytic viruses with ipilimumab can increase the effectiveness of cancer therapy.^[^ ^156^, ^157^ ^]^ Clinical trials in this field showed that combining T‐VEC with ipilimumab could effectively inhibit tumor growth and progression without any notable adverse effects in patients with melanoma.^[^ ^15^, ^158^ ^]^ A multicenter, open‐label, phase II study reported that a combination of oncolytic coxsackievirus A21(V937) with ipilimumab was associated with systemic immune activation, and most responses were durable and more than 26 weeks in patients with unresectable stage IIIC or IV melanoma. Moreover, this therapy was safe and controllable toxicities.^[^ ^159^ ^]^ G47Δ, a third‐generation oncolytic HSV‐1, was used in combination with anti‐CTLA4, and findings showed that antitumor responses were improved via recruitment of effector T cells into the TME and decreasing the frequency of Tregs at the tumor site. In addition, the expression of various genes involved in inflammatory responses and T cell activation was upregulated following the combination therapy.^[^ ^160^ ^]^

Influenza A virus (IAV), a promising oncolytic virus modified by reverse genetics and a recombinant influenza virus expressing CTLA4 (RFlu‐CTLA4) antibody, was constructed to treat hepatocarcinoma (HCC) in a subcutaneous H22 mouse model. This study showed that rFlu‐CTLA4 repressed tumor cell growth and prolonged the OS of treated mice.^[^ ^161^ ^]^ Moreover, rFlu‐huCTLA4 is another engineered IAV with the same function, which can efficiently replicate in various cells and have selective cytotoxicity in HCC cells than in normal liver cells. Intratumoral injection of rFlu‐huCTLA4 condensed tumor growth in vivo and prolonged the survival of mice. Furthermore, the number of CD4^+^ and CD8^+^ T cells was pointedly augmented in the treated tumor‐bearing BALB/c mice.^[^ ^162^ ^]^ It has been revealed that following systemic administration of oncolytic virus M1, the number of infiltrated Tregs in the TME was increased in melanoma and prostatic tumor models. CD25^high^ Tregs could suppress CD8^+^ T cells. Targeting Tregs using anti‐CTLA4 antibody combined with M1 oncolytic viruses induced tumor suppression and lymphocytotoxicity via decreasing Tregs and increasing the infiltration of CD45^+^ immune cells CD4^+^ or CD8^+^ T cells, releasing IFN‐γ, perforin, and granzyme. Furthermore, this regime extended the OS rate compared with monotherapy with M1 in the mentioned tumor models.^[^ ^163^ ^]^

Based on the available evidence, combining oncolytic viruses with immune checkpoint blockers creates synergistic responses that can be evaluated and confirmed by increasing the infiltration of antitumor cells in the tumor site and releasing antitumor cytokines such as IFN‐γ.^[^ ^164^ ^]^ Therefore, increasing the number of oncolytic virus pipelines and the development of immune checkpoint blockers may create a revolution in cancer therapy in the future.

#### 6.2.3 Triple Therapy

Another attractive therapeutic approach is triple therapy (anti‐PD1/PD‐L1+anti‐CTLA4+oncolytic viruses), which effectively activates immune memory and inhibits cancer recurrence more than dual therapy.^[^ ^165^ ^]^ An investigation used triple therapy by combining oncolytic adenoviruses with anti‐PD‐L1 and anti‐CTLA4. This study showed that this triple therapy inhibited tumor growth and prolonged survival in a TNBC model by recruiting CD8^+^ T cells, inducing memory T cells, and decreasing the number of Tregs and TAMs (M2 phenotype) in the TME.^[^ ^166^ ^]^ In another investigation, oncolytic HSV expressing IL‐12 (G47‐mIL‐12), anti‐CTLA4 antibody, and anti‐PD‐1 antibody were used to treat glioblastoma (GBM). Findings demonstrated that triple therapy was more effective than dual therapy using anti‐CTLA4 and anti‐PD‐1 antibodies in improving the survival of animals.^[^ ^165^ ^]^

These results indicate that triple therapies may be effective in cancer therapy because oncolytic viruses reprogram the immunosuppressive TME, whereas immune checkpoint inhibitors such as anti‐CTLA4 and anti‐PD‐1/PD‐L1 antibodies hinder immune escape upon virotherapy. However, these triple therapies require further study to determine the effectiveness of the treatment and possible adverse effects.

### 6.3 Combining of Oncolytic Viruses and CAR‐T Cell Therapy

Previous studies revealed that combining genetically modified oncolytic viruses with CAR‐T cells improved the infiltration of CAR‐T cells into the TME, enhancing the therapeutic outcomes of CAR‐T cells in solid tumors.^[^ ^167^, ^168^ ^]^ For instance, modified oncolytic viruses expressing cytokine and chemokine genes can promote the locomotion and recruitment of CAR‐T cells to the tumor tissue. Ad5D24/CCL5/IL‐15 is a genetically modified oncolytic virus encoding *CCL5* and *IL‐15* genes, and the intratumoral administration of this oncolytic virus could enhance the infiltration of anti‐GD2 CAR‐T cells into the TME, eliminating tumor cells.^[^ ^169^ ^]^ CCL5 and IL‐15 are involved in the locomotion and migration of NK cells to the tumor site, eliminating tumor cells.^[^ ^42^, ^170^ ^]^

Another modified oncolytic adenovirus induces the expression of IL‐2 and TNF‐α (OAd/IL‐2/TNF‐α) could also boost the functions of mesothelin‐redirected CAR‐T cells.^[^ ^171^ ^]^ It has been reported that combining modified oncolytic viruses expressing BiTE with CAR‐T cells can induce robust antitumor responses.^[^ ^172^ ^]^ Folate receptor‐α (FR‐α) is a potent target for CAR‐T cell therapy because it is highly expressed in several tumor cells.^[^ ^173^, ^174^ ^]^ However, due to the mechanisms of tumor cell escape by regulating the expression of FR‐α, monotherapy with anti‐FR‐α CAR‐T cells is ineffective. An investigation designed an oncolytic adenovirus expressing BiTE targeting EGFR in combination with anti‐FR‐α CAR‐T cells.^[^ ^175^ ^]^ This study demonstrated that combination therapy promotes CAR‐T cell proliferation, activation, and tumor cell killing ability and activation. Furthermore, the survival of studied mice was increased following the combination therapy.^[^ ^175^ ^]^

On the other hand, the expression of immune inhibitory molecules can affect the effectiveness of CAR‐T cell therapy.^[^ ^176^, ^177^ ^]^ Accordingly, researchers used oncolytic viruses encoding anti‐PD‐1/PD‐L1 antibodies to overcome this problem.^[^ ^178^, ^179^ ^]^ CAdVECPDL1, an oncolytic adenovirus expressing anti‐PD‐L1 mini‐antibody (mini‐body), was combined with anti‐Her2 CAR‐T cells. The findings showed that the antitumor effect of CAR‐T cells was significantly improved following the therapy. Additionally, the survival rate of the treated animals with CAdVECPDL1 and anti‐Her2 CAR‐T cells was prolonged compared with monotherapy by CAdVECPDL1 or anti‐Her2 CAR‐T cells.^[^ ^180^ ^]^ It seems that the use of oncolytic viruses expressing anti‐PD‐L1 mini‐bodies is preferable to the systemic administration of anti‐PD‐L1 antibodies because mini‐bodies have greater penetration power into tumor tissue with minimum adverse effects.^[^ ^181^ ^]^ Another study demonstrated that oncolytic adenoviruses expressing anti‐PD‐L1mini‐bodies and IL‐12p70 (CAd12/PDL1) could induce CAR expression and killing of tumor cells by CAR‐T cells in an HNSCC xenograft model.^[^ ^17^ ^]^ Recently, it has been revealed that combining oncolytic viruses encoding epitopes to stimulate naïve TCRs can enhance CAR‐T cell proliferation, activity, efficacy, and immune memory in mice models of melanoma and glioma.^[^ ^182^ ^]^ Therefore, combination therapy using oncolytic viruses encoding genes involved in antitumor responses or anti‐immune checkpoint antibodies in combination with CAR‐T cell therapy can be effective for cancer treatment.

Finally, it should be noted that in virotherapy, high‐dose administration of oncolytic viruses and combination with other anticancer approaches such as chemotherapy or immunotherapy will upsurge the possibility of off‐target toxicity.^[^ ^183^ ^]^ As discussed, employing a miRNA (de‐)targeting system may reduce the likelihood of off‐target replication without sacrificing oncolytic effectiveness. In this regard, an investigation controlled the replication of the measles virus with various miRTS positions such as N and L 3′ untranslated region in the measles virus genome to develop effective and safe cancer virotherapy.^[^ ^183^ ^]^

## 7 Concluding Remarks

Based on available knowledge and the outcomes of studies in recent decades, cancer monotherapy with various anticancer agents and methods has not been very successful, and combination therapy often leads to improved antitumor responses. Complex TME conditions, tumor escape tactics, T cell exhaustion, expression of inhibitory molecules and mediators, immunosuppressive cell infiltration in the TME, and tumor heterogeneity are challenges facing cancer treatment, and combination therapy can overcome these barriers to help treat cancer. Oncolytic viruses are no exception to this rule, and combining them with other immunotherapy‐based methods can cover the weaknesses of each method and improve the strengths. On the other hand, triple therapies using virotherapy and anti‐checkpoint antibodies can also be attractive options in cancer treatment because they simultaneously inhibit different pathways and reinforce antitumor responses and tumor elimination. However, this type of treatment's safety and side effects are not yet fully understood, and further studies are needed in this area.

## Conflict of Interest

The authors declare no conflict of interest.

## Authors Contribution

H.Z., X.‐Z.M., and B.Z. designed the study and took responsibility for the integrity of the data and the accuracy of the data analysis. H.Z. has made substantial contributions to the study concept and design. H.Z. wrote the first draft of the article and prepared the figures. All authors contributed to the interpretation of the data and writing of the article and approved the final version of the article.
